# Factors supporting resilient performance and mental well-being among health care professionals in home care settings: a qualitative study

**DOI:** 10.1186/s12913-025-13917-w

**Published:** 2026-01-02

**Authors:** Teklay Tesfay Kidanemariam, Maren Kristine Raknes Sogstad, Siri Wiig, Cecilie Haraldseid-Driftland

**Affiliations:** 1 SHARE-Centre for Resilience in Healthcare, Faculty of Health Sciences, Department of Quality and Health Technology, University of Stavanger, Mailbox 8600, Stavanger, 4036 Norway; 2 Centre for Care Research, The Norwegian University of Science and Technology, NTNU in Gjøvik, P.O. Box 191, Gjøvik, 2802 Norway

**Keywords:** Elderly care, Health care professionals, Home care, Resilience, Resilience in healthcare, Resilient performance, RiH theory, S4R, Support4Resilience

## Abstract

**Background:**

The growing demand for home care services, mainly due to an aging population and increasing complexity of care, has intensified the shortage of qualified health care professionals, resulting in increased workloads, emotional strain, a constant need to adapt to changing care demands, and poor mental well-being. It is therefore crucial to understand what helps healthcare professionals to stay in their roles and perform under pressure. This study aimed to explore the factors that support resilient performance and mental well-being among healthcare professionals in home care settings.

**Methods:**

This study used a qualitative exploratory design with a thematic analysis approach. Seven semi-structured focus group interviews were conducted with 34 participants working in three home care settings in three Norwegian municipalities. Data were collected through semi-structured interviews using an interview guide that focused on actual work situations, resilience in healthcare, patient safety, mental well-being, leadership support, and engagement in care services.

**Results:**

Four themes were extracted: *collaborative culture*, describing collective problem-solving, team unity and collegiality; *leadership support practices*, highlighting the leadership accessibility, supportive, and attentive to the professional and emotional needs of Health care professionals; *organization of work*,* roles and procedures*, highlighting the structured routines, tools and technologies, as well as *individual coping and adaptation strategies*, describing Health care professionals’ flexibility and task prioritization in handling unpredictable situations.

**Conclusion:**

A dynamic interplay of individual factors and organizational elements shaped healthcare professionals’ ability to cope with and adapt to the challenges and stressors encountered in home care settings. Our research contributes to the growing understanding of the importance of actively promoting collaborative team cultures, accessible leadership, clear organizational structures, and supportive coping strategies in developing resilient and sustainable home care services.

**Supplementary Information:**

The online version contains supplementary material available at 10.1186/s12913-025-13917-w.

## Background

Over the past few decades, home-based care has become a rapidly growing part of healthcare in Norway and many other Western countries [1–3]. This is mainly due to a growing elderly population living with chronic and often complex health conditions [4, 5]. In addition, the heightened focus on user-centered care, the advancement of new support technologies, and widespread efforts to enhance responsiveness, continuity, efficiency, and equity in healthcare systems are all contributing to the growth [5, 6].

Projections suggest that the number of care-dependent older people will increase alongside noncommunicable diseases as the leading cause of chronic illness and disability [6]. In Norway, the proportion of people aged 80 and older is projected to constitute nearly 10% of the population by 2060 [7]. Across the EU, the share of individuals in this age group has been increasing in recent years, reflecting a broader trend of population aging [8].

Furthermore, home care environments are becoming increasingly complex and demanding, requiring healthcare professionals working in these environments to assume a wide variety of roles, supporting patients not only in clinical and curative care but also in supportive, rehabilitative, palliative, and comforting care [2, 9, 10]. They are thus expected to carry out these responsibilities in unpredictable, often resource-limited conditions.

The increasing demand for home care services, combined with the rising complexity, has exacerbated the shortage of qualified healthcare professionals. In Norway, estimates suggest a shortage of around 13,000 registered nurses by 2030 [11]. The gap in the EU is estimated at 4.1 million by the same year [8, 12]. High turnover rates further worsened this shortage, leaving healthcare professionals under psychological stressors such as workload, time pressure, and physically demanding tasks [4]. These factors present substantial challenges to the mental well-being of healthcare professionals and their ability to deliver high-quality care. Hence, it is essential to develop and implement strategies that strengthen the capacity and sustainability of healthcare professionals in home care settings, ensuring sound working conditions and enhancing their mental well-being. An important step towards this goal is understanding what helps healthcare professionals stay in their roles and continue delivering quality care under demanding, complex conditions.

Resilience in Healthcare (RiH) has been operationalized through the concept of Resilience Engineering (RE), which focuses on enhancing the capacity of healthcare systems and professionals to adapt, respond, and maintain high-quality care under varying and often challenging conditions [13]. In this context, resilient performance refers to the outcome of achieving four resilience capabilities within the system: anticipating disruptions, monitoring the system, responding to demands, and learning from experience [14].

While the general definitions of resilience often focus on individuals or organizations, most previous research has focused on individual resilience, particularly among hospital staff during acute events such as the COVID-19 pandemic [15–21]. However, in line with the RiH perspective, resilience is conceptualized as a system-level property that emerges from interactions among people, teams, and organizational processes and is enacted through four interrelated potentials: anticipating, monitoring, responding, and learning [22, 23]. The RiH framework extends the four potentials into ten capacities that describe how resilience is enacted in everyday healthcare practice: responding, monitoring, anticipating, learning, coordinating, aligning, leadership, sensemaking, adapting, and coping [24, 25].

In our study, resilience incorporates the broader theoretical understanding and the more specific RiH conceptualization centered on the ten resilience capacities. Therefore, we define resilience as the capacity to adapt and maintain performance under changing conditions [22], aligning with the general understanding of resilience as the ability of individuals or organizations to bounce back or adapt after adversity [26].

Research on these resilience capacities has largely been confined to hospital settings [27, 28], leaving limited knowledge of how they manifest in home care, where work is more variable, distributed, and less controllable [2, 9, 10, 29–31]. This highlights the pressing need to explore what supports resilience specifically in this setting.

Resilience has also been associated with positive mental well-being outcomes among healthcare professionals, including lower stress and burnout, and higher job satisfaction and work engagement [32]. In the current study, we conceptualize mental well-being as the psychological and emotional health of home care professionals, encompassing both negative outcomes (e.g., stress, emotional demands, risk of common mental disorders) and positive states (e.g., meaningful interpersonal relationships and job satisfaction) [33].

Building on this, the current study investigates how healthcare professionals understand and enact resilient performance in Norwegian home care, drawing on the ten capacities defined in the Resilience in Healthcare framework [24, 25]. Furthermore, we investigate how these capacities appear in everyday home care practice, which factors support them, and how they relate to the mental well-being of health care professionals.

Specifically, three research questions guide this study:

1. What factors contribute to resilient performance?
2. What factors contribute to mental well-being among healthcare professionals?
3. How do factors associated with resilient performance and mental well-being interact?

### Contextualizing the study

#### The support4 resilience project

This study is part of the larger Horizon Europe-funded Support4 Resilience (S4R) project 2024–2028 (Project ID: 101136291) [34]. Focusing on elderly care provision across various healthcare settings, including public and private nursing homes, home care services, and hospital-to-home care, the S4R project aims to develop a research-based, innovative platform known as the S4R toolbox. This toolbox is designed to help healthcare leaders phase out ineffective practices, thereby freeing up time and resources for more effective approaches. It also promotes greater autonomy among healthcare professionals and supports learning from positive outcomes. The toolbox provides a range of cost-effective tools, both individual and collaborative, to help implement effective resilience and mental well-being strategies.

Involving 14 partners from seven countries, Australia, Finland, Italy, the Netherlands, Norway, Romania, and Spain, the project entails cross-country data collection, toolbox development, implementation, process evaluation, cost-effectiveness and effectiveness evaluations, theory development, and policy recommendations.

This article is part of the initial investigations of the S4R project. By exploring the factors that contribute to resilient performance and mental well-being among healthcare professionals from the Norwegian home care context, it aims to provide a foundation for developing the S4R toolbox.

#### The home care setting in Norway: work organization and daily practices

The Norwegian healthcare system is publicly funded and governed at the national, regional, and local levels [35]. The Ministry of Health and Care Services (MoHCS) oversees national-level policy, while provision is decentralized to Regional Health Authorities (RHAs) and municipalities [36]. RHAs are responsible for specialist care, whereas municipalities organize and finance primary care, including home care, the front line of the Norwegian healthcare system [37].

The way home care is organized and practiced vary across countries [38]. In Norway, home care is predominantly publicly owned, managed, and delivered by the country’s 357 municipalities, which, as of 2025, have a population ranging from approximately 5,000 to 500,000 [39].

Although service provisions should be tailored to individual needs based on assessments, the tasks include providing medical assistance, nursing care, practical support to maintain daily activities, rehabilitation, and end-of-life care at patients’ homes [40]. In Norwegian home care, nurses do not typically perform practical assistance tasks such as cleaning or meal preparation. These activities are handled by other home-care personnel or by private or separate municipal providers [41, 42].

While basic home care services are, in principle, free of charge, users share the cost for additional services, such as practical assistance (e.g., home cleaning) and access to senior citizen centers, if needed [43].

While organizational models vary between municipalities, home care is generally team-based, with defined roles, planning systems, and digital care plans. Healthcare professionals typically rotate shifts, including weekends, with most providing care in patients’ homes, and traveling from one residence to another, while some members work in institutional facilities.

Generally, a home care team begins their shifts by reviewing patient lists, sharing care information, and distributing work assignments in a scheduled meeting. The daily tasks in home care settings include basic care responsibilities (hygiene, dressing, and meals) and advanced care responsibilities (medication administration, wound care, catheterization, and ostomy care).

Nurses constitute the core workforce of home care (including elderly care) services in Norway, with most holding bachelor’s degrees and some serving in advanced practice roles at the master’s level. As of 2023, approximately 25% of the 122,000 registered nurses work in elderly care [44, 45]. In addition to nurses, healthcare assistants (in Norwegian: *Helsefagarbeidere*) receive training equivalent to a two-year postsecondary vocational degree and comprise approximately 91,000 workers in the same year [44]. To meet service needs, untrained or in-training assistants are also employed temporarily.

## Methodology

### Study design

A qualitative exploratory design [46] was used in this study. Seven focus group interviews were conducted across three home care settings in Norway. Given the study’s aim, focus group interviews are well-suited for collecting rich experiential data [47] to gain insight into the factors that enable healthcare professionals to manage challenges, sustain high-quality care, and support their mental well-being in everyday home care practices.

### Research setting and recruitment of research participants

The study was conducted in three municipalities in eastern Norway, representing urban, suburban, and rural areas. Their geographical areas range from approximately less than 1,000 km² to over 1,000 km². Their populations range from less than 10,000 to more than 20,000 inhabitants [48].

Site recruitment was conducted through the municipalities. Municipal leaders were initially contacted, and they assisted by connecting the research team with the leaders of home care services.

In total, 34 healthcare professionals were recruited, including registered nurses (*n* = 16), healthcare assistants (*n* = 17), and one care assistant (see Table 1).

The majority of participants were from Municipality 1, with 24 participants (8 nurses, 15 healthcare assistants, and one care assistant). Municipality 2 contributed 5 participants, all of whom were nurses. Municipality 3 contributed 5 participants (three nurses and two healthcare assistants.

### Data collection

A semi-structured interview guide comprising 23 questions organized into seven topic areas was developed specifically for this study in collaboration with the Support4Resilience (S4R) consortium. The guide was initially developed in English (see Supplementary File 1) and was later translated into Norwegian, the participants’ native language, to ensure clarity and facilitate better understanding [49].

The interview guide covered the following topics: general information about the participants’ professional background, education, and training; actual work situations (Work as done); resilience in healthcare; patient safety; mental well-being; leadership support; and Informal caregivers’ engagement in care services. No questions were asked regarding person- or patient-sensitive issues.

Two researchers conducted focus group interviews in November 2024 at prearranged meeting places in home care settings. One of the researchers is an associate professor of care research with substantial knowledge of quantitative and qualitative research methods. The other is a PhD candidate with a solid understanding of qualitative research methodology. The researchers had no prior contact with the study participants.

In total, seven focus group interviews were conducted: five in Municipality 1, one in Municipality 2, and one in Municipality 3. Six of the focus groups included five participants each, while one included four. Apart from their availability and willingness to participate in the study, no additional recruiting criteria were applied.

During the mixed focus group interviews, it was observed that in some groups, nurses contributed more frequently to the discussion than the other participants. These observations were noted in field notes to support contextual information of the data collected.

Each focus group interview session lasted approximately 90 min and was audio-recorded via the *Nettskjema Diktafon* mobile app, a data collection tool that enables the ethical and secure storage of qualitative data [50]. Prior to each interview session, participants received both written and oral information about the study aim, and written informed consent was obtained from all participants.

This study employed the Consolidated Criteria for Reporting Qualitative Research (COREQ) checklist as a reporting framework [51].

### Characteristics of the participants

Table 1 shows that the study included 34 participants, of whom the majority were women (*n* = 31), including healthcare assistants (*n* = 17) and nurses (*n* = 16). Three of the 17 healthcare assistants were men. Nearly all the participants (*n* = 33) held permanent positions.

In terms of participants’ years of work, 9 participants had less than 5 years, 11 had 6–10 years, 8 had 11–20 years, and 6 had more than 21 years.

**Table 1:** Characteristics of the participants

	*N* = 34
Gender	
Women	31
Men	3
Professional background	
Nurses	16
Healthcare assistants (*Helsefagarbeidere*)	17
Care assistant	1
Years of work	
Below 5 years	9
6 to 10 years	11
11 to 20 years	8
21 years and above	6
Work situation	
Permanent	33
Temporary (needs based)	1

### Data analysis

The first author transcribed the interviews *verbatim* [52] and imported the transcripts into NVivo 1.61 [53] for analysis. The data analysis for this study was conducted according to Braun and Clarke’s six-step framework for thematic analysis [54] (see Table 2).

**Table 2:** Braun and Clark’s six-step framework is followed in the data analysis process

Phase	Description
Data familiarization	Each participant’s interview transcript was transcribed, read multiple times, and annotated with field notes to gain a deep understanding of the data.
Generating Initial Codes	Codes were created systematically for each participant’s transcript to capture key features and meaningful statements. Initial coding yielded **nine** preliminary themes across all participants.
Searching for Themes	The preliminary themes were grouped based on patterns, similarities, and relationships across cases to form tentative themes and subthemes.
Reviewing Themes	Themes and subthemes were refined through cross-case analysis, discussions, and iterative review to ensure they accurately reflected the data.
Defining and Naming Themes	After refinement, **four themes** and **eight subthemes** were finalized through consensus among the authors.
Writing up the findings through a coherent narrative.	A clear, structured account of the themes, data extracts, and analytic commentary was written to present the findings.

Several strategies were employed to ensure the trustworthiness of this qualitative study. The first author conducted initial data coding, followed by regular debriefing sessions with co-authors to review, validate, and refine the emerging themes, reducing individual bias and enhancing interpretive depth [55]. Credibility was further strengthened by the fact that two researchers conducted each interview and met afterward to compare, discuss, and note emerging patterns. Dependability was enhanced through auditing and documenting analytical decisions and coding changes [56]. While member checking was not formally conducted, triangulation across interview data and observational notes taken during the interviews by the researchers helped reinforce the credibility of the findings [57]. Descriptions of the study context, participant characteristics, and home care settings enhanced the study’s transferability, enabling readers to assess its applicability to similar contexts [58].

## Results

This study explored the factors that healthcare professionals in home care settings perceive as supporting resilient performance and mental well-being. Using the thematic analysis approach, we identified four main themes and eight subthemes (see Table 3), each illustrated with representative quotations.

**Table 3:** Overview of themes (*n* = 4) and subthemes (*n* = 8)

Main themes	Sub-themes
1. Collaborative Culture: Working and Learning Together	1. Sharing knowledge and workloads 2. Team unity and collegiality
2. Leadership Support Practices	1. Accessible and engaging leadership 2. Autonomy: both demanding and rewarding
3. Organization of work, Roles and Procedures	1. Documentation as a communication and coordination tool 2. Support systems, technologies, and tools
4. Individual Coping and Adaptive Strategies	1. Adaptability during unpredictable challenges 2. Reducing stress through task prioritization

### Theme 1: Collaborative culture: working and learning together

The theme *Collaborative Culture* captures aspects related to healthcare professionals’ perceptions of collective problem-solving and knowledge sharing (i.e., the exchange of practical insights) within their roles, as well as the overall workplace atmosphere in home care settings. Although home care work often involved physically working alone, participants emphasized that they rarely felt isolated. Healthcare professionals reported a strong sense of team collaboration and continuous informal and formal learning, which were cultivated through daily interactions. Shared responsibility for patient care was noted as a defining feature of both structured and spontaneous collaboration in home care work.

In some mixed-focus group interviews, nurses were dominant in providing examples that shaped this theme. This dynamic likely influenced which aspects of collaboration were emphasized in the findings.

### Sharing knowledge and workloads

Participants perceived that a strong collaborative culture contributed to both professional development and care quality, even in settings where healthcare professionals primarily worked independently. Participants reported that despite each HCP having individual tasks for the shift, the nature of their work always made them feel part of a team, both by sharing practical knowledge and by supporting each other with workloads:

Even though we work alone with our lists, we are not alone—we are part of a team. We work together, and we are good at helping each other. If someone finishes early, we start calling around and offering help, picking up visits from others’ lists. (M1, P3)

When performing their daily activities, healthcare professionals needed to remain responsive to unexpected patient needs, exchange expertise, and provide both emotional and practical support as needed. In this context, both planned and unplanned interactions, such as shift handovers, mid-shift check-ins, and informal conversations, were seen as valuable opportunities for mutual learning and growth. Informal learning often occurred spontaneously as healthcare professionals shared experiences, reflected on challenging cases, and passed along practical knowledge throughout their day. In addition to supporting learning from experience, these practices also contributed to anticipation, another instance of resilient performance and adaptive capacity.

Breaks and shared lunch times also became arenas for peer exchange. While some participants noted efforts to limit work-related conversations during lunch, many acknowledged that discussions about patient care and professional challenges naturally continued around the lunch table.

Participants described learning as embedded in day-to-day routines, with experienced staff mentoring others and providing guidance on complex or unfamiliar tasks. In addition to addressing challenges as they arose, this culture of peer support and ongoing learning was perceived to enable healthcare professionals to share experiences, clarify uncertainties, and enhance their competence. *“… unexpected things always come up, so we must discuss them continuously”* (M3, P5), *“If we are unsure about something, like lifting or transferring patients, we might do a mid-shaft check-in, where we provide some training.”* (M1, P4)

Additionally, collaboration extended beyond the immediate care team. Interdisciplinary collaboration with occupational and physical therapists was described as providing healthcare professionals with valuable insights into managing physically demanding tasks, such as patient lifting and movement. By conducting joint patient visits and assessing patient needs together, they offered practical tips that participants felt enhanced both patient care and their workday, especially when tasks became physically challenging.

### Team unity and collegiality

Participants frequently described their workplace as open and supportive compared to other workplaces they were familiar with. The positive team atmosphere was perceived to foster collegiality, making the workday more enjoyable. It was often noted that it was acceptable to feel uncertain about something; when in doubt, one could simply ask, and support was readily available. This openness fostered strong interpersonal relationships and encouraged a collaborative culture.

Unity and collegiality were described as the foundation of psychological safety. The presence of experienced staff members appeared to reinforce this environment, fostering continuity and trust that sustained a healthy team culture. *“What works well here is that we have unity and good collegiality. This is important because people like to feel safe around each other.”* (M2, P4)

### Theme 2: Leadership support practices

This theme encompasses participants’ perceptions of leadership in terms of accessibility, supportiveness, and responsiveness to their professional and emotional needs. Open communication, mutual trust, and a supportive leadership presence were recognized as key elements that contributed to day-to-day functioning and to psychological safety, autonomy, and professional growth among the healthcare professionals.

#### Accessible and engaging leadership

Several participants used phrases such as ‘the leader is visible’ and ‘the door is always open’ to describe the perceived engagement and responsiveness of leadership in the daily activities of the home care setting. Most noted that they could approach their leaders without needing an appointment, provided that the leader was not preoccupied with other tasks or busy on the phone. Many appreciated the ability to approach their department leaders informally when they met them at work. This accessibility and responsiveness were perceived to allow healthcare professionals to raise concerns, ask questions, and seek guidance without facing barriers.

I actually feel that I am being heard and that I can talk to [the leader] with my shoulders down without feeling like I will be judged for the mistakes I make and what I think…. if I have any ideas about things that should be changed and so on and yes, generally being heard and feeling safe when I speak is important for me… (M1, P16)

The participants also favored an open approach that included anonymous ‘post-it’ notes as an alternative for those who were less comfortable with direct communication. Participants valued this as a simple, non-intimidating way to communicate with their leaders.

Participants frequently linked supportive, present leadership to fostering a positive work environment and promoting teamwork in home care settings. Leaders were praised for their swift responses (especially during high-pressure situations), which participants felt built trust and confidence.

A participant recalled, *“There was an incident, and [the leader] handled it incredibly well; it happened in the blink of an eye, and she gained my trust that way”>* (M1, P3)

Leaders’ involvement in frontline tasks was viewed as a crucial aspect of engaging leadership, particularly during periods of increased workload. This became especially evident during busy times, such as the summer holiday season, when staff shortages due to vacations placed additional strain on home care teams. Participants emphasized that leaders who adopted a hands-on approach and actively supported staff in the field not only helped alleviate the immediate workload but also appeared to foster stronger team cohesion.

#### Autonomy: Both demanding and rewarding

Most participants valued autonomy in their work, describing it as both rewarding and demanding because they were responsible for making independent decisions. On the one hand, it allowed them to tailor their choices to patient needs and build confidence through independent problem-solving. On the other hand, this autonomy came with pressure, as healthcare professionals were solely responsible for their assessments and actions.

Over time, this independence was perceived to contribute to a sense of competence and adaptability, as reflected in the following statement: *“When no one is around to ask, I have to make the decision myself. It is scary at first, but it makes me stronger and more confident in my decisions.”* (M3, P4) This suggests that while leadership inaccessibility could pose challenges, it also reinforced participants’ sense of autonomy and their ability to develop resilience in their roles. 

Other participants noted that the system clarified decision-making boundaries, which was described as fostering a sense of security and psychological safety. This clarity helped healthcare professionals reduce uncertainty, as they knew when to act independently and when to seek support. *“We also have a good system in place; I clearly understand which decisions I can make on my own and which ones need to be referred to either the head nurse or the leader.”* (M3, P1)

### Theme 3: Organization of work, roles, and procedures

This theme explores how structured organizational routines, clearly defined roles, and the effective use of technological tools and documentation were perceived by participants to contribute to the delivery of high-quality, consistent care. Participants explained that organizational clarity regarding roles, responsibilities, procedures, and expectations in home care settings enabled more predictable workflows, reduced uncertainty, minimized stress, and supported a more stable work environment.

#### Documentation as a communication and coordination tool

Healthcare professionals stressed that accurate, up-to-date documentation is more than an administrative requirement in home care; it is a critical component of patient safety, care coordination, and the overall patient experience. Documentation was described as an essential component of internal communication, vital for tracking care processes, recording clinical interactions, and ensuring the continuity of care. *“We read the report from the past shift every day when we start work for all the patients we have. We spend at least five to ten minutes on it. That way, you can see what has been done and what still needs follow-up with the patients.”* (M2, P5) 

Participants also reported that routine reviews of patient records were essential to ensuring continuity and accountability, particularly during shift change or when staff returned from time off. Writing detailed notes was viewed as an expected responsibility, crucial for maintaining awareness of recent developments and preventing information gaps in patients’ care histories.

Clear and thorough record-keeping was particularly valued in situations requiring coordinated responses, such as initiating new clinical procedures or patient-specific interventions. This was seen to facilitate ongoing evaluation and knowledge sharing among the healthcare professionals.

Documentation was also regarded as a critical tool for accountability, particularly when patients responded to questions or concerns from their family members. *“[…] when the leader receives questions about a patient, she can check the report, see what has happened and who wrote what, and then explain the details*.*”* (M1, P1) 

#### Support systems, technologies, and tools

Participants outlined how they use various systems and tools to provide structure and enable continuity of care. This includes task distribution systems, alarms, electronic messaging systems, medication dosing systems, and standardized procedures. Collectively, these systems and tools were described as helping staff stay organized, anticipate tasks, and maintain consistent workflows, even during periods of high demand.

Technological tools, including medication dispensers, sensor-based alarms, smart cameras for safe and secure follow-up, door and bed alarms, and emergency call systems, were reported to be used daily to increase patient safety and workflow efficiency. However, not all systems were equally effective across contexts. For example, the limited accessibility of the procedure portal while in the field due to a lack of remote access was seen to limit its usefulness during home visits. This was noted by one participant: *“The tools make our workflow smoother and safer, but some systems only work well when we’re at the office. For instance, sometimes we can’t get into the procedure portal while we are out in patients’ homes, so we have to rely on our memory or call someone instead.”* (M1, P12)

In addition to digital tools, participants utilized analog methods, such as reminder notes and checklists, which they frequently reviewed at the beginning of each day to stay organized and prioritize tasks.

Participants indicated that while tools and routines were essential for the smooth operation of home care, assigning dedicated staff to manage operational tasks was considered crucial for ensuring care continuity and efficiency. The shift from rotating responsibilities to a more stable staffing arrangement was perceived as enhancing both the efficiency and continuity of care services in home care settings. *“Initially, we rotated responsibilities, she would sit one day, I would sit next, and someone else the third. Now, there is more consistency: one person handles everything full time on weekdays, including electronic messages, phone calls, hospital messages, and ongoing communication.”* (M1, P3) 

The results also revealed that a new task distribution system, which defined clear areas of responsibility for each person, had been introduced to clarify roles and responsibilities. While this system was generally viewed as a positive development, promoting structure, accountability, and more efficient workflows, participants’ experiences varied. Some participants believed it improved coordination and reduced ambiguity in daily tasks, while others felt it limited flexibility or created challenges when tasks overlapped or required teamwork across role boundaries.

### Theme 4: Individual coping and adaptive strategies

This theme encompasses the personal and professional competencies that healthcare professionals use to maintain their mental well-being and perform under pressure. When faced with unpredictable challenges that test their emotional regulation and high workloads, participants reported employing a variety of adaptive strategies, including staying calm and flexible, prioritizing tasks, navigating complexity, protecting team functioning, and continuing to deliver care.

#### Adaptability during unpredictable challenges

Adaptability was identified as a core professional competency essential for maintaining performance in the face of unexpected situations, complex patient needs, and limited resources. Participants highlighted that flexibility was crucial at both the individual and team levels, allowing healthcare professionals to support one another and perform professionally despite unpredictable conditions.

Working alone, especially during staff shortages or on weekends, requires healthcare professionals to assess unfamiliar scenarios and respond independently and quickly. For example, unfamiliar equipment, such as infusion pumps, necessitated on-the-spot problem-solving and self-directed learning. This ability to adapt was perceived as essential for sustaining professional performance in challenging situations.

Staying calm and regulating one’s pace were described as essential stress management strategies that healthcare professionals employed to prevent stress from escalating within the team. As one respondent reported: *“… We are good at de-escalating stress… If one person starts panicking, it can spread quickly, but we know how to manage that. It is almost like a skill—keeping things calm and handling the unexpected.”* (M1, P1) 

This flexibility also applied to caring for patients with complex mental health needs, requiring real-time adjustments in approach and communication.

#### Reducing stress through task prioritization

Healthcare professionals shared that they prioritized tasks based on their critical nature during high-pressure shifts. While participants acknowledged the importance of person-centered care, they also need to focus on more critical tasks, such as administering medication or providing personal hygiene assistance, at the expense of less critical interventions, including vitamin administration or nonessential visits, to manage workloads and reduce stress. One participant states it as follows: *“You want to do everything for everyone, but sometimes, you simply can’t. You have to decide what is essential and what can be postponed.”* (M1, P17)

When the schedule was tight, healthcare professionals adjusted their schedules, often postponing time-consuming tasks until later in the day, when their workload allowed for greater flexibility. In other cases, healthcare professionals substituted physical visits with check-in calls, especially when the patient’s needs were not urgent. *“Sometimes you need to rethink how you deliver care. If I know a patient is stable, I can make a quick call instead. It’s not ideal, but it gives me the time I need for the more critical tasks.”* (M1, P9)

Though most agreed that decisions were necessary to ensure quality care for those in more critical need, several noted the emotional complexity of prioritization and the discomfort of facing criticism for delays or changes in plans. *“It can be challenging to face criticism for our priorities, especially when some complain while others accept waiting a bit longer without saying anything. This is part of our everyday work.”* (M1, P2).

## Discussion

The overall aim of this study was to explore the factors that healthcare professionals in home care settings experience supporting their resilient performance and mental well-being.

Guided by the RiH theory, which focuses on maintaining stability while adapting to both expected and unexpected challenges within the healthcare system [59], we identified four key interrelated factors: *Collaborative Culture*,* Leadership Support Practices*,* Organization of work*,* roles and procedures*,* and Individual Coping and Adapting Strategies* (see Table 3).

### Factors that support resilient performance

Our findings suggest that organizational and contextual factors, such as a strong collaborative culture, leadership, autonomy, and peer support, are key factors to how healthcare professionals manage daily demands and support one another emotionally in their work. In this respect, team solidarity was described as enhancing both individual and collective resilience and helping the team members navigate the multifaceted challenges of home care with a shared sense of purpose [60]. This finding is consistent with earlier research, which shows that social cohesion, open communication, and trust-based teamwork reduce occupational stress, build resilience, and support healthcare professionals’ overall well-being, which are fundamental to adaptive capacity [4, 61, 62].

While many of these dynamics have been studied in the context of the COVID-19 pandemic, the underlying principles of team cohesion and adaptive capacity remain highly relevant to the day-to-day work of home care. The nature of home care is often unpredictable, with professionals working in decentralized settings and frequently managing complex situations independently. However, this context continues to demand strong collaboration and flexibility. To strengthen our understanding of how these dynamics function specifically in home care, more research is needed that is situated directly within this practice setting.

In this study, both planned and spontaneous interactions throughout the workday, such as quick check-ins, case-related discussions, and impromptu problem-solving, were found to play important roles in maintaining team cohesion while enabling continuous learning. Participants described that these exchanges also help them anticipate potential disruptions in patient care by sharing early signals and practical insights. This collaborative culture reflects two key resilience capabilities: learning from experience and anticipating emerging needs in home care practice [62, 63]. It also illustrates a key feature of resilient healthcare’s adaptive capacity, in which teams dynamically reallocate attention and resources in response to changing demands [22].

This form of situational responsiveness reflects what can be described as *contextual resilience*, an ability to draw on tacit knowledge [64], learn quickly in the moment, and rely on professional judgment to navigate resource gaps. These adaptive skills enable healthcare professionals to stabilize challenging operational situations and maintain a sense of professional competence.

Participants in our study described collaborations with colleagues and seeking help to solve problems and complete tasks as a normal part of their routine and something that was encouraged behavior to do so as part of their daily work. This finding aligns with the resilience framework by Lyng et al. (2022) [25], which identifies behaviors such as seeking help and mutual engagement among team members as highly beneficial for improving their practices. As such, involvement, one of the ten capacities for resilience in healthcare, plays a central role in sustaining the functionality and responsiveness of healthcare teams [25].

Our result also underscores the value of ‘slack’ in organizational routines [65], where time spent on discussing and addressing work tasks enabled learning, emotional processing, and real-time coordination among healthcare professionals. In a home care environment, where healthcare professionals often work autonomously with little or no immediate support, these seemingly minor moments of peer interaction play a crucial role in supporting the resilience and adaptability of healthcare professionals.

Accessible and supportive leadership practices were also identified as a significant influence on team resilience. When leaders were approachable, responsive, and actively engaged in frontline tasks during periods of high demand, participants interpreted this as a sign of solidarity and shared responsibility. Participants also noted that these leadership practices helped foster trust and psychological safety within the team, conditions that are essential for resilient performance in high-pressure care environments [66]. This finding aligns with previous research, which demonstrates that leadership practices rooted in openness and support can have a significant impact on team functioning and the capacity to adapt during challenging situations [60, 67–69]. Similarly, in their research on teamwork in healthcare during COVID-19, Anjara et al. [60] found that frontline engagement by clinical leaders marked by shared tasks, collective leadership, and psychological safety supported stronger teamwork and organizational citizenship behaviors.

In addition, our study found that workplace autonomy functions as a dual dynamic, both empowering and potentially stressful, depending on the clarity of role boundaries. This finding reflects the distinctive nature of home care, where professionals often work in patients’ homes, with less direct supervision than in institutional settings [4], a context that may demand continuous adaptive capacity to sustain resilient performance. Our study found that care professionals reported feeling more confident and secure in their roles when their areas of authority and responsibility were clearly defined. The main reason for this was that this kind of clarity strengthened their sense of autonomy, often encouraging professional growth and flexibility. However, participants also noted that this independence could also lead to uncertainty and added stress when guidance or organizational support was lacking. These findings align with earlier research, which has shown that the unpredictable, autonomous nature of home care nurses’ work necessitates independent decision-making, which can be both empowering and demanding, continually testing healthcare professionals’ resilience [70].

This leads us to another, but related, finding of the study. The way work was organized, along with clearly defined roles and routines, emerged as a crucial factor in supporting the resilient performance of healthcare professionals. In this context, the use of tools and technologies, such as medication dispensers, alarm systems, emergency call systems, and digital monitoring devices, was viewed as essential for enhancing patient safety and for a more efficient, consistent delivery of care.

The need for mobile access to clinical decision-making tools, such as electronic patient portals and real-time documentation systems, was another crucial element found to enhance efficiency and support informed decisions, both in the field and in on-site primary care settings [71].

Healthcare professionals found that these tools and technologies reduce the need for frequent in-person visits to patients’ homes while maintaining surveillance and response capabilities. Similarly, previous studies have shown that the use of technological tools enhances medication adherence, facilitates timely responses to emergencies, and supports staff performance by reducing the need for constant physical presence while maintaining high standards of care and surveillance [62, 72–74].

### Factors that support mental well-being

Healthcare professionals in home care settings employ various personal strategies to maintain their mental well-being and sustain performance despite the inherent variability and uncertainty of home care work. Adaptability emerged as a core competence across participants’ narratives and was described as a distinct technical skill by itself that also reduces psychological strain. Participants highlighted the importance of responding flexibly to unpredictable situations, such as unfamiliar clinical scenarios or high-pressure encounters, which helped them avoid feelings of helplessness or a sense of loss of control. The ability to independently “figure things out”, especially during weekend shifts when immediate support was often unavailable, was seen as essential for preventing the escalation of stress and for allowing individuals to maintain confidence and emotional stability in the face of complex home-based care demands. This finding aligns with prior qualitative research on home health nurses, which indicates that autonomy, problem-solving, and adaptability are essential to their daily practice, particularly in situations that require swift judgment and carry significant responsibility [75].

Previous studies have indicated that adaptive capacity in healthcare, such as coping, aligning, reframing, and innovating, is an essential ability for reducing emotional overload and enhancing psychological endurance in dynamic care environments, showing adaptive capacity functions as a mental well-being resource [76].

In addition to personal flexibility, our study revealed the importance of collective stress regulation in supporting mental well-being. When team members actively intervened to de-escalate an overwhelmed situation, they helped interrupt stress cycles before they intensified. These interactions reflect team-level adaptive capacity, often grounded in psychological safety and a shared sense of responsibility [62], both of which are known contributors to mental well-being because they mitigate isolation and normalize seeking support [77].

The findings indicate that although healthcare professionals are expected to manage their emotional responses individually, they operate within a relational context where colleagues routinely step in to offer support. This interplay between personal coping and collective responsiveness suggests that resilience is not solely an individual attribute; rather, it emerges as a dynamic, shared process reinforced through daily interactions, peer support, and collaborative learning, thereby sustaining mental well-being and preventing the erosion of individual coping resources [63, 77]. This reciprocal responsiveness contributes to psychological safety and shared responsibility, both of which are central to adaptive team functioning [78].Consistent with our findings, previous studies have shown that strong communication and team-based collaboration can reduce burnout, enhance psychological resilience, and improve care quality [2, 25, 79–82].

The participants also described regulating their work speed as a method to handle stress. Having autonomy over time management allowed healthcare professionals to maintain control over their workflow, even when this meant shorter breaks or longer work hours, a known determinant of mental well-being [83]. Despite operational constraints, healthcare professionals, in this study, maintain their commitment to person-centered, holistic care while regularly adjusting their work priorities to meet the individual needs of their patients. This finding aligns with that of Norlyk et al. (2019) [75], who observed that home care nurses constantly balance efficiency demands with a strong professional ethos of individualized care, often stretching time to preserve care quality.

Task prioritization, particularly under staffing shortages, further illustrates how coping strategies intersect with mental well-being. In our study, essential care tasks, including medication administration and insulin delivery, as well as hygiene support, received priority over less essential services, such as housekeeping and routine check-ins. Healthcare professionals viewed these decisions as necessary adjustments to maintain essential care operations during constraints, such as during staff shortages. This finding aligns with broader research indicating that during periods of limited staffing or high workload, healthcare workers implicitly ration care, focusing on acute and safety-critical tasks while deferring fewer essential activities [84].

However, as some decisions may lead to patient dissatisfaction or criticism, participants of this study found the prioritization process problematic and emotionally complex. This finding aligns with previous research, which suggests that deferring less critical services can lead to moral distress, particularly when patients express dissatisfaction or feel neglected [85]. Another study on moral distress in home-care nursing found that prioritizing tasks under resource constraints often triggered ethical tension and emotional strain [86].

From a resilience and patient safety perspective, these decisions reflect the adaptive trade-offs described in resilience engineering, where practitioners continually adjust their performance to balance competing demands to maintain overall system performance and safety [13, 87–89]. This may suggest that in home care settings, such trade-offs involve not only stress management but also the maintenance of ethical and safety standards in challenging situations.

The finding of this study shows that individual coping and adaptation strategies, such as task prioritization and peer support, are essential. However, for these efforts to be successful, the roles and expectations must be clearly established. Further, individual strategies should be combined with systemic support approaches for enhancing both workplace resilience and the mental well-being of healthcare professionals in home care settings [90].

The findings of the current study have multiple implications for practice. To support the resilience and mental well-being of healthcare professionals, healthcare leaders should consistently engage with staff through regular check-ins and feedback mechanisms and actively participate in frontline work. These measures signal shared responsibility and solidarity.

Furthermore, healthcare leaders can cultivate a collaborative team environment by establishing clear roles and responsibilities within their settings. To achieve this, leaders can create open communication channels, foster reflection on challenges and successes, build a culture of shared decision-making, and facilitate peer mentoring. Additionally, leadership support, regular feedback, and recognition can also cultivate collaboration and create an adaptive environment among healthcare professionals. In addition, recognizing and promoting the everyday importance of peer support can strengthen social cohesion, buffer stress, and enhance adaptive capacity in home care settings.

In addition, ensuring access to practical digital tools can reduce administrative burden and enhance information sharing among team members. These tools may help decrease unnecessary cognitive and emotional workload, which is essential for maintaining mental well-being.

Our findings underscore that resilient performance and mental well-being are closely intertwined rather than separate phenomena. Organizational structures, leadership practices, collaborative culture, and individual coping strategies collectively enhance both adaptive performance and mental well-being. Recognizing resilience as a relational, context-dependent process helps explain how daily interactions, peer support, and systemic resources jointly sustain mental well-being in home care settings.

### Strengths and limitations

The qualitative design was well-suited to the research question, likely enabling an in-depth exploration of healthcare professionals’ experiences and perceptions regarding the factors contributing to positive outcomes in their everyday practices [91]. Using focus group interviews likely enriched the data by capturing more nuanced insights through the group’s dynamics and interactions [92]. The interview guide was developed in collaboration with the S4R consortium, incorporating diverse expert perspectives, which likely enhanced its content validity [93].

The involvement of municipal leaders in participant recruitment may have helped foster trust among participants, contributing to the richness and reliability of the data [94]. Moreover, the inclusion of registered nurses, healthcare assistants, and one untrained care assistant provided a heterogeneous pool of participants, enabling the capture of diverse perspectives on the factors that support resilient performance and mental well-being. The study’s adherence to the COREQ checklist [51] likely ensured transparency and methodological rigor in design, data collection, and reporting.

However, some important limitations exist in this research that need to be considered for a thorough interpretation of the findings. Although the qualitative approach used likely enabled in-depth exploration of the study’s aim, it may also introduce subjectivity into data interpretation [91]. The recruitment method through leadership might have attracted more engaged and accessible participants, potentially introducing selection bias [95].

Furthermore, although the study was conducted across three home care settings in three municipalities in the eastern part of Norway, the majority of participants came from a single site, potentially skewing the data toward that context. This uneven distribution may have influenced the data. Nonetheless, given their specific nature, we have described the contextual conditions to enable others to assess the relevance of our results for their healthcare systems.

Additionally, only one care assistant and three male participants were included, which may limit the applicability of the findings. Although females are generally overrepresented in healthcare, making the participants representative of the actual workforce composition in this context, it still limits broader applicability. Lastly, although the interview guide was carefully translated into Norwegian, subtle interpretative differences cannot be ruled out [96].

The group dynamics during the interview process might be another concern. In mixed sessions with nurses and healthcare assistants, nurses were observed dominating the discussion. This may likely limit healthcare assistants’ contributions. Although these patterns were taken into account during the analysis, they may still have shaped the diversity and depth of the data.

Finally, the predominantly positive accounts of the participants may reflect a few dynamics. First, although participants acknowledged various challenges, the aim of our study was to identify what supports positive adaptive capacity and mental well-being in home care settings. Hence, participants naturally focused on examples of what works well in their workplaces. Second, the natural tendency to view one’s own work environments favorably might have also played a role. Additionally, though participation was voluntary, we cannot rule out the possibility that the recruitment process attracted more engaged or motivated staff, which, in turn, could have further shaped the overall positive responses.

## Conclusion

In this study, we investigated the factors that support resilient performance and mental well-being among healthcare professionals working in Norwegian home care settings. Key themes: a *collaborative culture*,* supportive and accessible leadership practices*,* clearly structured work organizations*,* including roles and procedures*, as well *as individual coping and adaptation strategies*, help healthcare professionals in home care settings cope with daily demands, reducing stress levels while improving their job satisfaction.

As home care continues to face challenges with workforce sustainability and poor mental well-being among its staff, our study provides valuable insights into how these elements can be used to develop and implement strategies that support individual and organizational resilience, thereby ensuring sound working conditions and enhancing the mental well-being and retention of healthcare professionals.

The study demonstrates that home care settings require a comprehensive approach that combines organizational elements with individual factors to promote resilient performance and mental well-being.

## Supplementary information

Below is the link to the electronic supplementary material.

Supplementary Material 1

## Data Availability

The interview data used for this study can be made available from the corresponding author upon reasonable request, provided that permission is obtained from the study participants.

## Abbreviations

MoHCS: Ministry of Health and Care Services
RiH: Resilience in Healthcare
S4R: Support4Resilience
