# Supplementary material for: Factors supporting resilient performance and mental well-being among health care professionals in home care settings: a qualitative study
Source: BMC Health Serv Res. 2026 Jan 2;26:39. doi: 10.1186/s12913-025-13917-w (PMC12784549; doi:10.1186/s12913-025-13917-w)
Supplement: Supplementary file 1 — Supplementary Material 1 [file 12913_2025_13917_MOESM1_ESM.pdf]

Focus Group Interview Guide: Healthcare Professionals.

General pointers to the interviewer

- By workers we mean personnel who work at the frontline providing care for elderly patients; this could be all types of paid staff such as nurses, care assistants, physiotherapist, etc.
- The semi structured approach provides the interviewer with the opportunity to ask follow-up questions and include new questions if required.
- For the majority of the questions, we seek understanding and descriptions of their actual day-to-day work and not how they would like to perform the work if they had more resources/time etc.

Semi-structured guide

General information
Aim: *to seek information of their background and current position to set the scene.*
Pointers to the interviewer: *All questions need to be answered by all participants in the focus group (go round the table making sure each worker answers questions 1-4).*

1) What is your professional background (education, training)?
2) What type of position do you have?
3) For how long have you been working in elderly care?
4) How is your position structured? Do you work closely with your leader? Do you work in a team?

Work as done:
Aim: *to seek understanding of their everyday care work and tasks.*
Pointers to the interviewer: *focus on getting descriptions of their actual day-to-day work and tasks and not how they would like to perform if they had more resources, time, etc.*

5) Briefly describe your **work situation and typical work tasks** (e.g., roles, responsibilities, type of patients cared for, etc.)?

Resilience in Healthcare:
Aim a: *to seek descriptions of what they believe contributes to the provision of high-quality care and what and why positive outcomes occur in their everyday practices.*
Aim b: *to seek information of what type of adaptations that take place to maintain services when facing challenges, changes, pressure, and variations.*
Pointers to the interviewer: *Resilience in healthcare emphasizes the importance of understanding and learning from what goes well in the organization. Examples of factors that can contribute to positive outcomes are competence, technology, people and financial resources. The term adaptations is similar in meaning to terms like adjustments, transformations, reorganizations, and workarounds. Use the term(s) best understood by the informants. Ensure that the informants talk about what resources they currently have available to them and not what they would like to have. The focus here is mainly on everyday work, however they might also talk about how they have handled more critical situations like Covid-19, which is also valuable to know about.*

6) **What works well** in your day-to-day work? What are you/is your unit good at and why?

7) What **contributes to the provision of quality** care for elderly in your unit (what type of solutions, practices, resources like e.g., technology, people, knowledge and information improve everyday work).
8) What types of **adjustments/workarounds/adaptations** do you use to handle challenges (e.g., lack of resources, peak situations), changes (e.g., in patient conditions, deterioration, patient flow) and variations (e.g., patient needs, team composition, available resources, competence) in your day-to-day work?
9) How are adjustments/workarounds/adaptations initiated? Agreed upon among colleagues in your unit or individually initiated?

Patient safety:
Aim: *to seek understanding of common adverse event/near misses/everyday events that happen in their workplace, how they foresee such events, and what they do to avoid them happening. Furthermore, we are interested in information on how they prepare for crises and adverse events.*

10) What are common **adverse/serious events, near misses and everyday events** in your workplace, and how can these be mitigated?
11) Do you perceive your unit/organization **to have an atmosphere** where staff can **openly discuss** adverse/serious events, and if so, what factors contribute to having an open atmosphere?
12) How does your unit/organisation **prepare for crises and adverse events**?

Mental wellbeing:
Aim: *to explore current challenges and stress factors for workers in their day-to-day job.*
Pointers to the interviewer: *These questions concern the worker as an individual, and <u>not</u> at the organizational level.*

13) What type of **challenges** (e.g., technological, resources, workflow, information exchange, competence, etc.) do you have to deal with in your day-to-day work?
14) What type of **stress** factors (e.g., emotional, ethical, workload, time management, etc.) do you encounter in your day-to-day work?

Leadership support:
Aim: *to seek information of how and when staff experience support from their leaders.*
Pointers to the interviewer: *Particular themes of interest are what is done to achieve psychological safety within their work environment and how leaders facilitate for staff involvement in decision making.*

15) What **kind of leadership support** (individual, structural, relational, organizational, etc.) is provided for **staff in your unit**? (Could be e.g., confirmation, acknowledgement, mentoring, education and training opportunities, meetings arenas, informal gatherings, etc.)
16) To what extent are staff involved in **decision making**?
17) How are the **ideas, solutions, and feedback** that staff provide received by your leader?
18) Do you have **opportunities for professional development,** and if so, what kind of training do you get?
19) How do you view your **career prospects** at your workplace?

Informal caregivers:
Aim: *to understand how involvement of informal caregivers in service provision and decisions related to care is facilitated by the staff, and how other types of stakeholders are involved in the provision of care.*

Pointers to the interviewer: *Of particular interest are approaches to involving informal caregivers in service provision and care decisions, and how, when and why this happens. Examples of other types of stakeholders might be local organizations (e.g., cancer organizations, the Red Cross) or interest groups (e.g., groups providing activities for elderly).*

20) How are **informal caregivers** involved in care in your unit/organization? In what ways can involvement of patients and informal caregivers be strengthened in your unit?
21) Do you involve **other stakeholders** (e.g., local organizations and interest groups) in providing quality care for the elderly in your unit and if so, how?

Overall:
Aim: *To obtain information on support, resources, and solutions perceived to improve their work situation.*
Pointers to the interviewer: *Ensure that the informants talk about what resources they currently have available to them and not what they would like to have when describing suggested support, resources, and solutions.*

22) Could you give three suggestions for how your current work situation could be improved? The more specific the better.
23) Is there anything else you would like to add?
